# *A Reply to* Mr. Cruikshank'*s Observations in Defence of the New System of Chemistry; in a Letter from* Dr. Priestley *to* Dr. Woodhouse, *Professor of Chemistry in the University of Pennsylvania*

**Published:** 1802-10-01

**Authors:** J. Priestley

**Affiliations:** Northumberland


### *A Reply to* Mr. Cruikshank's *Observations in Defence of the New System of Chemistry; in a Letter from* Dr. Priestley *to* Dr. Woodhouse, *Professor of Chemistry in the University of Pennsylvania.*

[ From the Medical Repository, edited by Drs. Mitchell and Miller, and published at New York. ]

Dear Sir,

Though you are an advocate, at least in part, for the new theory of chemistry, the principles of which I controvert, your candour is such, that I wish to address to you what I have to reply to the observations of Mr. Cruikshank on the subject of the experiment with *finery cinder and charcoal*, which you repeated in a manner that does you the greatest credit.

These two substances, though heated separately till no air or vapour of any kind can be expelled from them, yet when, immediately after this, they are heated together, they yield a large quantity of air, a great proportion of which is inflammable; and the new theory, which excludes phlogiston, requires that this inflammable air be a component part of water, which, of course, must be found in one or other of the two substances. Mr. Berthollet thinks it was contained in the charcoal, notwithstanding the heat to which it had been exposed. You allow, with me, that it was in the finery cinder, though we differ with respect to the decomposition of it. But Mr. Cruikshank has suggested a very different hypothesis; maintaining that water is not at all necessary to the production of this inflammable air; asserting that metals and their calces, in a very high temperature, have the power of decomposing fixed air, and that, in this case, the fixed air comes from the oxygen in the finery cinder, and the carbon in the charcoal.

After repeating my original experiment, which he found to be just, he did the same with the calces of other metals, particularly those of zinc, copper, lead, and manganese, and then concludes, that "in all these cases the air must come from the partial decomposition of the carbonic acid by the calx, when raised to a high temperature." But the inference that I think is more naturally drawn from them, is, that all these calces contain much water, and little or nothing else. This I have shewn to be the case with respect to several of them, especially that of zinc, and I doubt not but that some small portion of oxygen may be contained in them all. Indeed, we cannot absolutely say that any substance can be wholly expelled from any other with which it has been intimately combined by any process.

Before Mr. Cruikshank admitted that iron, or its calx, when raifed to a high temperature, can decompofe carbonic acid (fixed air) in this experiment, he fhould have tried whether it would do it in any other. If in any cafe, I fhould think it would do it when it was heated in this air by a burning lens, by which a greater heat can be produced than in any open fire. But this I found not to be the cafe, either with iron or this calx of it. In the laft fummer I went through a courfe of experiments with this view; but I always found fixed air not to be decompofed by this means; though I found that a portion of this air, and alfo of all the other kinds of air that are readily imbibed by water, was rendered immifcible in water by means of *heat*, reflected either from the calx of any metal, a piece of earthen crucible, or any other fubftance on which I threw the focus of the lens, when it was furrounded by this kind of air, confined by mercury or water. This, however, was no decompofition of the air, as there was no oxygen found in it after the procefs. The addition of permanent air was always *phlogisticated.*

Mr. Cruikfhank thought that if this heavy inflammable air came from the decompofition of the carbonic acid by the calx, he fhould fucceed better by employing *iron filings* in the place of finery cinder, "as they would have a greater affinity with oxygen;" and with this view he heated them together with a quantity of "common *chalk*, previoufly expofed to a low heat for ten minutes." From this mixture he procured a great quantity of air, and he thought that the acid (i. e. fixed air from the chalk) was decompofed by the iron; whereas, when he ufed well burned lime he got little or no air.

What I infer from this experiment is, that the chalk not being perfectly calcined, contained fome water as well as fixed air; and that this water, uniting with the phlogifton of the iron, formed the inflammable air that he found. Water I fuppofe to be the bafis of all kinds of air, and many fubftances retain it in any degree of heat. Chalk I have found to do it after long expofure to the heat of a fmith's forge.

Admitting the fixed air procured in the experiment with the finery cinder and charcoal to come in part from the oxygen in the finery cinder, how is this oxygen to be expelled from the calx, fince heat will not do it? And there is no inftance, I believe, in chemiftry, in which, when heat alone will not expel any conftituent part of a fubftance, it can be effected without the aid of an *affinity*, in confequence of which fome other fubftance takes its place. But here, according to the new theory, nothing is fuppofed to take the place of the oxygen in the finery cinder, fince it takes nothing from the charcoal, the iron being revived by the mere expulfion of the oxygen which made it a calx.

Mr.

Mr. Cruikſhank lays great ſtreſs on the difference that he found in the air that he procured in theſe proceſſes, from that which is got from charcoal and water. But I have obſerved that there is a conſiderable difference in the qualities of heavy inflammable air, not only according to the ſubſtance from which it is procured, but in the ſucceſſive ſtages of the ſame proceſs. He will find that I have examined this kind of air, as procured from a great variety of ſubſtances made to paſs in the form of vapour through hot earthen tubes, and in various other ways, and have given the analyſis of them. I always found that the firſt portion from charcoal was loaded with fixed air, but that, in the courſe of the proceſs, this diſappeared, the air burning with a lambent flame, and that towards the end it approached to the exploſive kind, ſuch as is obtained from the metals by acids.

I alſo found that more or leſs fixed air is procured by the decompoſition of heavy inflammable air by means of dephlogiſticated air; and though the air procured from finery cinder and charcoal ſhewed no ſign of its containing any mixture of fixed air (nothing of the kind being diſcoverable by lime-water), yet when it was decompoſed I found much more than the weight of the air, ſo that it could not have been previouſly contained in it in a ſtate of ſolution, but muſt have been formed by the union of the oxygen in the dephlogiſticated air, and the phlogiſton in the inflammable air.

That charcoal, uniting with water, ſhould give fixed air as well as inflammable air, I account for by ſuppoſing, what is by no means improbable, that this ſubſtance contains the elements of both the kinds of air, and that they want nothing but water to enable them to take the form of air.

On the whole, I think it is evident that Mr. Cruikſhank finds fixed air in circumſtances in which it cannot be formed, and makes it to be decompoſed by ſubſtances which have no ſuch power.

Submitting theſe obſervations to your better judgement,

I am, dear Sir, your's ſincerely,

J. PRIESTLEY.

*Northumberland,*

*November* 21, 1801.

---

*To the Editors, on the same Subject.*

GENTLEMEN,

I Lately ſent you, in a letter to Dr. Woodhouſe, a Reply to the Obſervations of Mr. Cruikſhank, in anſwer to one of my arguments arguments in favour of the doctrine of phlogifton, which he fays you will be fo obliging as to infert in your next number. I have, fince that, received a letter from a correfpondent in Paris, in which he informs me that the chemifts in that city boaft greatly of thofe obfervations of Mr. Cruikfhank, and fay that I muft now give up the controverfy: I therefore beg, that to that letter to Dr. Woodhoufe you would pleafe to add this.

I am not a little furprized that fuch excellent chemifts as the fupporters of the new theory in France unqueftionably are, fhould make fo great account of the aid of a perfon who has abandoned the moft effential part of their fyftem, viz. the neceffity of water to the formation of inflammable air. Mr. Lavoifier, treating of the inflammable air from charcoal and water, which is fimilar to that from charcoal and finery cinder, fays, in his "Elements of Chemiftry," p. 87 of the Englifh tranflation, that "it cannot be difengaged from the charcoal, and muft confequently be produced from the water." According to the new theory, the union of the oxygen, which is fuppofed to come from the finery cinder, with carbon, from the charcoal, can only form fixed air, and no kind of air that is inflammable. Mr. Cruikfhank muft therefore abandon the new theory in order to fupport his peculiar hypothefis.

If I do not receive a better defence of the new theory from its able fupporters in France, I fhall foon conclude that it is incapable of any juft defence, and that, as becomes ingenuous men, they will abandon it, as Mr. Cruikfhank has actually done. I am, Gentlemen, your's, &c.

J. PRIESTLEY.

